# The role of lysosomes in cancer development and progression

**DOI:** 10.1186/s13578-020-00489-x

**Published:** 2020-11-18

**Authors:** Tao Tang, Ze-yu Yang, Di Wang, Xian-yan Yang, Jun Wang, Lin Li, Qian Wen, Lei Gao, Xiu-wu Bian, Shi-cang Yu

**Affiliations:** 1 Department of Stem Cell and Regenerative Medicine, Institute of Pathology and Southwest Cancer Center, Southwest Hospital, Third Military Medical University (Army Medical University), Chongqing, 400038 China; 2 Department of Hematology, Xinqiao Hospital, Third Military Medical University (Army Medical University), Chongqing, 400037 China

**Keywords:** Lysosomes, Cancer, Metastasis, Energy metabolism, Spatial distribution

## Abstract

Lysosomes are an important component of the inner membrane system and participate in numerous cell biological processes, such as macromolecular degradation, antigen presentation, intracellular pathogen destruction, plasma membrane repair, exosome release, cell adhesion/migration and apoptosis. Thus, lysosomes play important roles in cellular activity. In addition, previous studies have shown that lysosomes may play important roles in cancer development and progression through the abovementioned biological processes and that the functional status and spatial distribution of lysosomes are closely related to cancer cell proliferation, energy metabolism, invasion and metastasis, immune escape and tumor-associated angiogenesis. Therefore, identifying the factors and mechanisms that regulate the functional status and spatial distribution of lysosomes and elucidating the relationship between lysosomes and the development and progression of cancer can provide important information for cancer diagnosis and prognosis prediction and may yield new therapeutic targets. This study briefly reviews the above information and explores the potential value of lysosomes in cancer therapy.

## Background

### Introduction to the lysosome

**Fig. 1:**
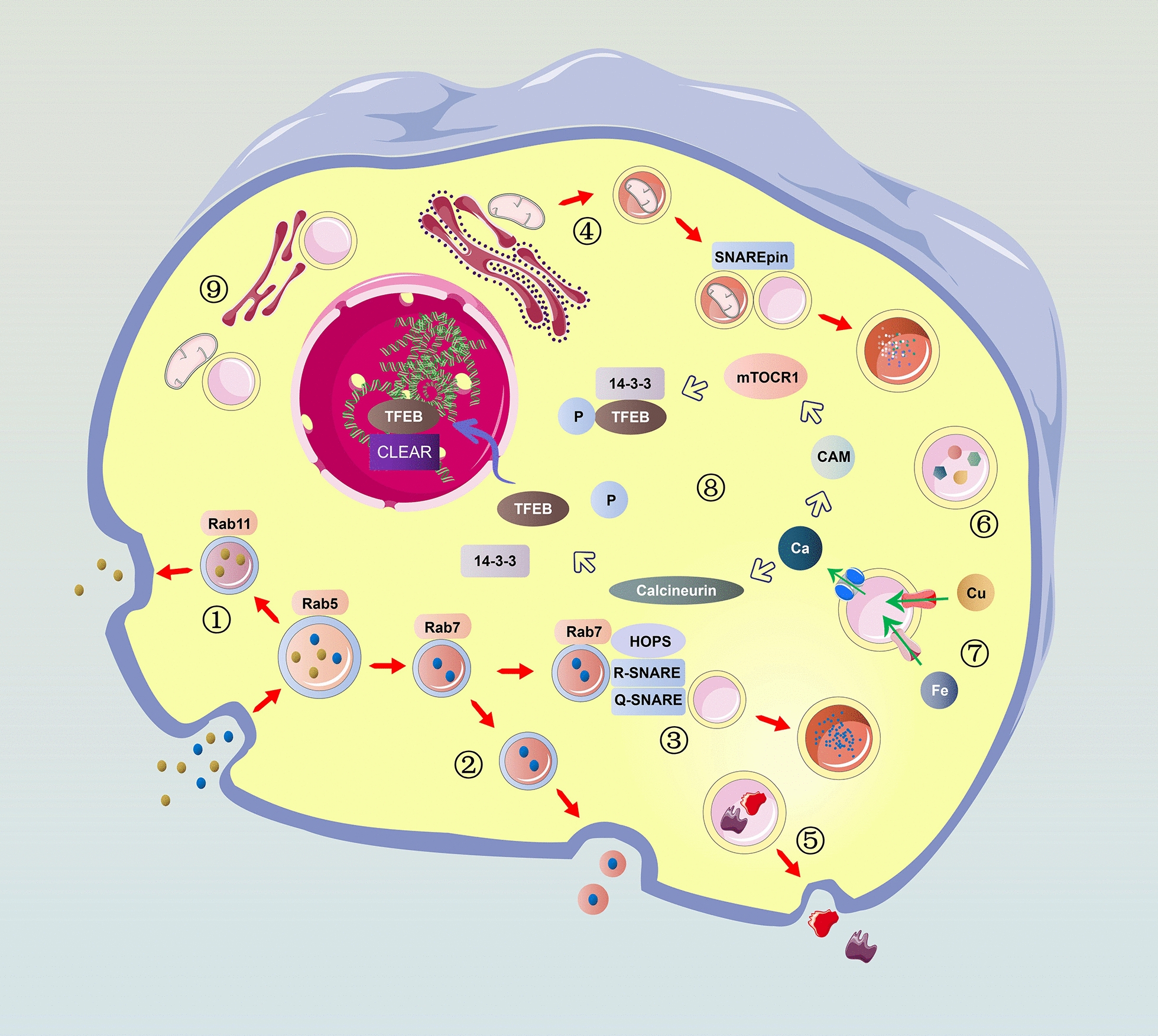
Lysosomes play a crucial role in intracellular transport. Vesicles formed by endocytosis and phagocytosis deliver cargo to Rab5-positive early endosomes. (1) Materials can be recycled to the plasma membrane by Rab11-positive recycling endosomes. (2) The remaining contents will be sequestered in Rab7-positive late endosomes, which can fuse with the plasma membrane to form exosomes. (3) Late endosomes can also fuse with lysosomes to degrade their cargo. During this process, Rab7 promotes the assembly of HOPS, which mediates lysosomal tethering with endosomes by pairing an R-SNARE on a lysosome (VAMP7 or VAMP8) with three Q-SNAREs on an endosome (syntaxin-7, VTI1b, syntaxin-8). (4) Lysosomal fusion with autophagosomes also requires SNAREs, including VAMP8, syntaxin-17 and SNAP29. (5) Lysosomes can also fuse with the plasma membrane to mediate membrane repair or discharge contents outside the cell, such as cathepsins or immune factors. (6) Lysosomes are the pools of metabolites in cells, including amino acids, sugars, lipids and nucleotides. (7) Metal ions are also stored within lysosomes. The storage of iron or copper can prevent their harmful accumulation in cells. (8) Lysosomal calcium channels, such as TRPMLs, can lead to the release of lysosomal calcium and activate mTORC1, which can phosphorylate TFEB and prevent TFEB nuclear translocation. TRPML1-mediated lysosomal calcium release can also dephosphorylate TFEB and promote its nuclear translocation and regulate lysosome biogenesis, autophagy, and lipid metabolism. (9) Lysosomes can form physical contacts with the ER, mediating the rapid transport of lipids, or with mitochondria, promoting mitochondrial fission or regulating the tricarboxylic acid cycle

**Fig. 2:**
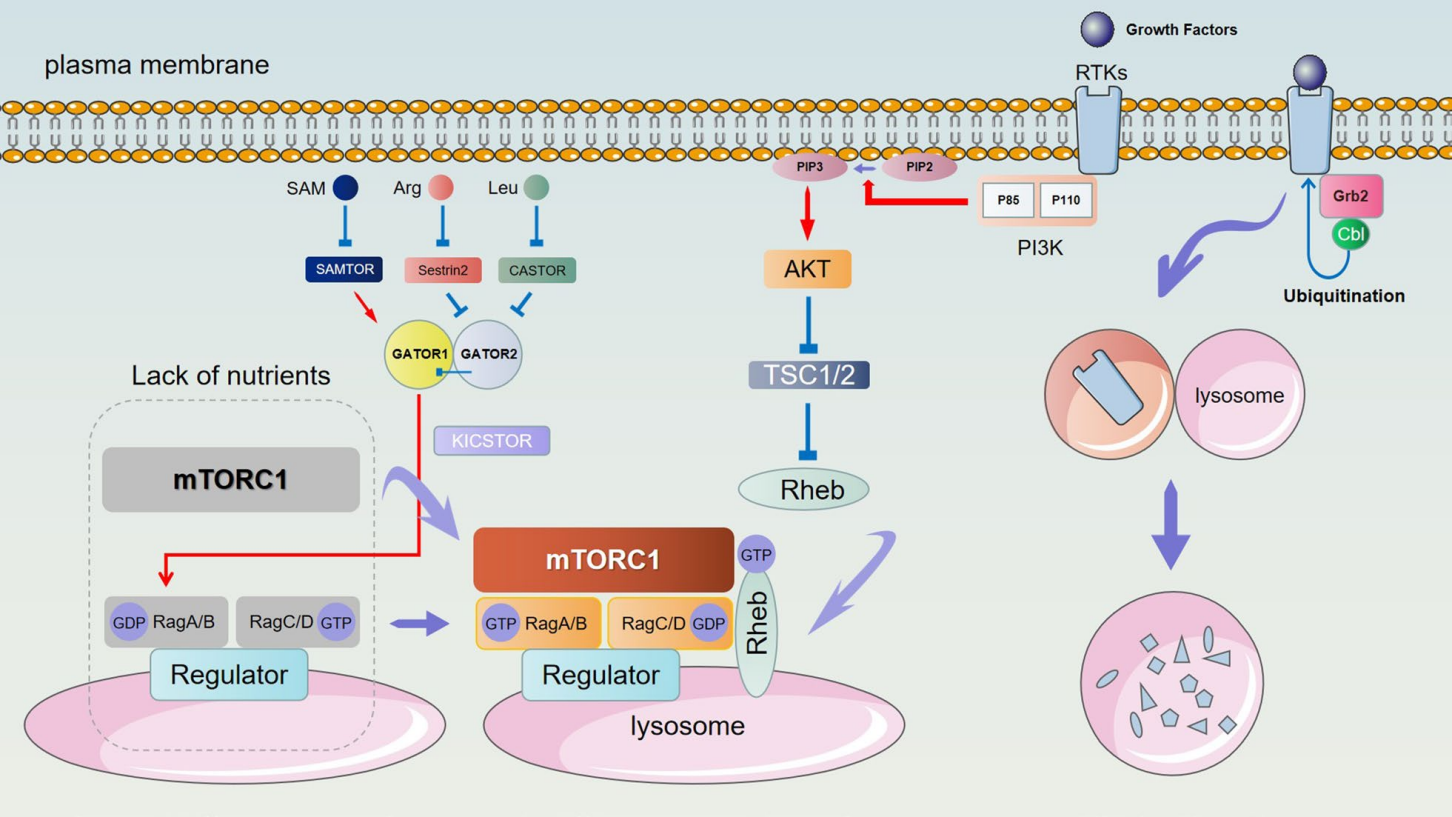
Lysosomes function as an intracellular signal transduction platform. The combination of growth factors and RTKs can activate the PI3K/AKT signaling pathway and negatively regulate TSC1/2, promoting Rheb to become GTP loaded, which can activate mTORC1. Rag GTPases are localized to lysosomes by Ragulator. When nutrients are lacking, mTORC1 is inactive in the cytoplasm, RagA/B is GDP loaded, RagC/D is GTP loaded, and Rag GTPases cannot bind to mTORC1. GATOR1 is a GAP for RagA/B, and its activity can be antagonized by GATOR2. Sestrin, CASTOR, and SAMTOR can sense Leu, Arg and SAM and interact with GATOR1/GATOR2. KICSTOR mediates GATOR1 recruitment to lysosomes and allows RagA/B to become GTP loaded and bind to mTORC1. Then, GTP-loaded Rheb unlocks mTORC1 kinase activity at the lysosome. Moreover, ligands binding to RTKs (e.g., EGFR) can recruit Grb2, which binds to Cbl. RTKs can be ubiquitinated by Cbl and trafficked to lysosomes for degradation

Lysosomes are an important component of the inner membrane system. This organelle was first discovered by Christian de Duve in 1955 and was so named because it contains a variety of hydrolases. Precursors of lysosomal enzymes are synthesized in the rough endoplasmic reticulum (rER) and then migrate to the cis-Golgi, where mannose residues on the oligosaccharide chain are phosphorylated to form mannose-6-phosphate (M-6-P), an important sorting signal for lysosomal enzymes. In the trans-Golgi network (TGN), phosphorylated lysosomal enzymes bind to M-6-P receptors, which direct the enzymes into clathrin-coated vesicles. Then, the clathrin lattice is depolymerized into subunits. The uncoated transport vesicles can fuse with autophagosome or heterophagosome to form autophagolysosome, heterophagic lysosome or phagolysosome. Lysosomes were previously believed to be the sites of the degradation of intracellular and extracellular substances. Therefore, researchers called lysosomes the “garbage disposals” of cells [1]. However, more in-depth studies showed this viewpoint to be too one-sided. Emerging evidence suggests that lysosomes may also be the cellular center for intracellular transport (Fig. 1), signaling (Fig. 2), and metabolism.

### Lysosomes play a crucial role in intracellular transport

The endosome–lysosome pathway is the primary means by which materials are trafficked and exchanged within the cell. Exogenous materials can be delivered to lysosomes by endocytosis and phagocytosis. Vesicles formed during this process transport their contents to early endosomes, which are Rab5 positive. Then, early endosomes become Rab7 positive. During this conversion, internalized materials can be recycled back to the plasma membrane in recycling endosomes, which are Rab11 positive. Meanwhile, the remaining contents can be stored in intraluminal vesicles (ILVs) in late endosomes, which are also termed multivesicular bodies (MVBs) [2, 3]. MVBs can move to the plus end of microtubules, mediated by kinesin, and fuse with the plasma membrane to form exosomes and secrete their ILVs. Alternatively, MVBs can move to the minus end of microtubules, mediated by dynein, and fuse with lysosomes to degrade their cargo. Many factors participate in the fusion of MVBs and primary lysosomes [4]. Small GTPases of the Rab family, particularly Rab7, can promote the assembly of tethering complexes (CORVET, HOPS), which mediate lysosomal tethering with endosomes. Tethering is followed by pairing of an R-SNARE (soluble N-ethylmaleimide-sensitive fusion attachment protein receptor) on the lysosomal membrane (VAMP7 or VAMP8) with three Q-SNAREs on the target membrane (syntaxin-7, VTI1b, and syntaxin-8). The four SNAREs, also termed SNAREpin, can bring the two bilayers sufficiently close for fusion to occur. Lysosomes can also discharge their contents outside the cell or mediate membrane repair by fusing with the plasma membrane [5, 6].

Moreover, lysosomes play an important role in autophagy. In mammalian cells, autophagy stimulation signals such as starvation can activate ULK complex (ULK1/2, FIP200, Atg13), which marks the initiation of formation of the phagophore. The recruitment of PI3K complex (VPS34, VPS15, Beclin1 and Atg14) follows, generating the endoplasmic reticulum domains called omegasomes, which contain PI3P. This process requires the participation of membranous vesicles containing Atg9. Omegasomes are highly curved regions and form autophagsomes by membrane expansion. This process requires the participation of two 2 ubiqutin-like conjugation complexes: Atg12-Atg5-Atg16L and LC3 [7]. After closure of the phagophores, the double-membrane autophagosomes mature and fuse with lysosomes. Under the mediation of dynein, kinesin, and MYO6, the autophagosomes and lysosomes move closer together [8]. Various molecules are involved in the fusion of autophagosomes and lysosomes, mainly including the following: the HOPS complex, Rab7, and adaptors that link autophagosomal or lysosomal components. The HOPS complex can interacts with autophagosomal Q-SNARE STX17 with the help of Atg14 [9]. Rab7 binds HOPS complex and promotes tethering [10]. The adaptor proteins, such as EPG5, TECPR1, and PLEKHM1, can bind to autophagosomal or lysosomal components, such as LC3 and ATG12–ATG5, while also interacting with the SNARE complex and/or core tethering factors (RAB7, the HOPS complex), thus ensuring the specificity of fusion [8]. The core machinery for fusion: SNAREs. A trans-SNARE complex, composed of autophagosomal STX17, SNAP29 and lysosomal R-SNARE VAMP8, can mediates the fusion of autophagosomes and lysosomes [11]. Upon fusion of lysosomes with the outer membrane of autophagosomes, the lysosomal contents enter the space between the two autophagosome membranes, and degradate the inner membrane in an LC3-dependent manner. Once autophagy is terminated, lysosomal membrane proteins are recycled from autolysosomes through tubular structures. At the tips of these tubules, nascent lysosomes, also named proto-lysosomes, are formed through a scission/budding process with the participation of KIF5B and DNM2 (dynamin 2) [8].

Lysosomes are pools of metabolites in cells that include amino acids, sugars, lipids and nucleotides. Metal ions are also stored within the lumen of lysosomes. Iron and copper storage within the lysosome can prevent their harmful accumulation in cells [12, 13]. Transient receptor potential mucolipin (TRPML) channels and two-pore channels (TPCs) are lysosomal calcium channels that can affect the functional status of lysosomes by regulating lysosomal calcium homeostasis; they also play important roles in regulating many other cellular processes [14]. The activation of TRPML1 leads to the release of lysosomal calcium, which can induce the association of calmodulin (CaM) with mTOR; this in turn activates the mammalian target of rapamycin complex 1 (mTORC1). The activation of mTORC1 can result in the phosphorylation of transcription factor EB (TFEB) and promote its interaction with 14-3-3, which then prevents TFEB nuclear translocation. Interestingly, TRPML1-mediated lysosomal calcium release can dephosphorylate TFEB and promote its nuclear translocation. TFEB can bind to the coordinated lysosomal expression and regulation (CLEAR) motif and regulate lysosome biogenesis, autophagy, and lipid metabolism. The activation of TPCs can also lead to TFEB dephosphorylation, which promotes its nuclear translocation. Furthermore, mTORC1 activation can block TPC activity [15].

Moreover, lysosomes can engage in physical contact with other organelles, such as the ER and mitochondria, by means other than membrane fusion. Within these contacts, the bilayers are maintained in close proximity by specialized tethering proteins. Some tethering proteins harbor specialized lipid-binding domains that can mediate the rapid transport of lipids [16, 17]. Contacts between lysosomes and mitochondria can promote mitochondrial fission or transport lysosome-derived metabolites into the mitochondrial matrix to fuel the tricarboxylic acid cycle [18–21].

### Lysosomes function as an intracellular signal transduction platform

Lysosomes play a crucial role in sensing nutrients and regulating cell proliferation and growth. When growth factors bind to receptor tyrosine kinases (RTKs), the PI3K/AKT signaling pathway is activated and negatively regulates TSC1/2 Rheb GAP (GTPase-activating protein), which promotes the GTP-loading of Rheb. When GTP-loaded Rheb is recruited to lysosomes, it will promote the activation of mTORC1 [22, 23]. Rag GTPases are anchored to the lysosomal membrane by Ragulator and V-ATPase proteins. When nutrients are lacking, mTORC1 is inactive and located in the cytoplasm; RagA/B is GDP loaded and RagC/D is GTP loaded, and thus, the Rag GTPases cannot bind to mTORC1 [24]. The GATOR1 complex is a GAP for RagA/B whose activity can be antagonized by GATOR2 [25]. In response to nutrients, mTORC1 is recruited to the lysosomal membrane. Nutrient sensors, such as Sestrin, CASTOR, SAMTOR and SLC38A9, can sense their ligands, leucine, arginine, S-adenosylmethionine and other amino acids in the lysosomal lumen and interact with the GATOR1/GATOR2 complex. Then, KICSTOR mediates GATOR recruitment to lysosomes and allows RagA/B to become GTP loaded [26]. Nutrient signals converge on Rag GTPases, which physically recruit mTORC1 to lysosomes, while growth factor signals converge on Rheb GTPase, which allosterically unlocks mTORC1 kinase activity at the lysosome [27]. The activation of lysosomal mTORC1 signaling can direct cell metabolism towards growth or promote quiescence and repair and inhibit the formation of autophagosomes. As mentioned above, mTORC1 also participates in the process of lysosomal calcium channel regulation of TFEB nuclear translocation.

Lysosomes can affect growth factor signaling through the endocytic degradation of growth factors, their receptors, or their signal transduction mediators [28]. Lysosomal degradation of RTKs plays an important role in regulating proliferation. Ligands that bind to epidermal growth factor receptor (EGFR) can recruit the adaptor protein Grb2, which binds to Cbl E3 ubiquitin ligase. EGFR is ubiquitinated by Cbl and then trafficked to the lysosome for degradation [28, 29]. Ligand binding can also recruit p70 and Clip4, which can interact with ubiquitinated EGFR to prevent lysosomal degradation. The same process can also control platelet-derived growth factor receptor (PDGFR) levels [30].

### Role of lysosomes in human disease

Lysosomes had been found to be involved in human diseases even before they were recognized as independent organelles. Mutations in genes encoding lysosomal-related proteins result in a family of diseases termed as lysosomal storage disorders (LSDs) [31]. LSDs are rare diseases, mostly inherited in an autosomal recessive manner. At present, more than 50 types of LSDs are known, and their pathogenesis is not yet fully understood. LSDs are characterized by the accumulation of digested products in the lysosomal lumen, such as amino acids, lipids, sugars and nucleotides [31]. The abnormal accumulation of digestion products in the lysosomal lumen will progressively disrupt its basic functions, including trafficking and the ability to fuse with autophagosomes, which in turn lead to neurodegeneration, metabolic imbalance and severe growth retardation [32, 33]. LSDs are usually classified according to their accumulated substrates and include sphingolipidoses, oligosaccharidoses, mucolipidoses, mucopolysaccharidosis, lipoprotein storage disorders, lysosomal transport defects, neuronal ceroid lipofuscinoses and other types. In addition, lysosomal dysfunction has been shown to be related to the pathogenesis and progression of Parkinson's disease, Huntington's disease and Alzheimer's disease, in which damage to the autophagy-lysosome system leads to impaired cell viability [32].

In recent years, much attention has been paid to the relationship between lysosomes and cancer. In addition to the degradation of substances, lysosomes are proposed to be involved in numerous cell biological processes, including intracellular pathogen destruction, plasma membrane repair, antigen presentation, cell adhesion/migration, apoptosis, metabolic signal transduction, exosome release, and gene expression regulation [1, 34].

These processes are all closely related to cancer development and progression. Existing studies have shown that lysosomes help cancer cells cope with environmental stress and participate in cancer development and progression mainly through the following aspects. (1) Lysosomes directly degrade nonessential macromolecules through the autophagy pathway [35]. During cancer development and progression, the demand for nutrients and energy by cancer cells increases. In the case of local ischemia and hypoxia, the autophagy pathway in cancer cells is activated to cope with a lack of sufficient nutrients. The hydrolases in lysosomes degrade various intracellular and extracellular macromolecules to recycle and reuse biological materials [36–38]. (2) Lysosomes regulate, transport and degrade cell surface molecules, such as cytotoxic glycoprotein T lymphocyte antigen-4 (CTLA-4), thus participating in the immune regulation of cancers [37, 39, 40]. (3) Lysosomes regulate intracellular and extracellular pH to ensure cancer cell survival [41, 42]. Due to aerobic glycolysis, the production of lactic acid in cancer cells is increased, resulting in an increase in the intracellular H^+^ concentration. The resulting acidic microenvironment seriously affects the activity of cancer cells. Proton pumps on the lysosomal membrane transport H^+^ into the lysosome lumen, thereby stabilizing the pH in the cytoplasm of cancer cells [41, 42]. (4) Lysosomes release cathepsin and “remodel” the extracellular matrix (ECM) [40, 43, 44]. Lysosomes can move to the plasma membrane, release various enzymes, such as cathepsin, to the outside of the cell and degrade collagen fibers and integrins to “remodel” the ECM and promote the metastasis of cancer cells [40, 43, 44]. (5) In cancer cells, MVBs can move to the plus end of microtubules and fuse with the plasma membrane to form exosomes and secrete their ILVs. Hence, exosomes are currently recognized as important mediators of cell-to-cell communication in cancer progression and metastasis [45]. It has been proposed that let-7 miRNAs play a tumor-suppressive role in targeting oncogenes such as RAS and that cancer cells can release let-7 miRNAs via exosomes to maintain their oncogenicity [46, 47].

Further studies have shown that the activity of multiple enzymes in lysosomes is significantly increased in many cancer tissues compared with paracancerous tissues [48], which suggests that lysosome functions are active. In addition, lysosome functions are associated with their spatial distribution. In cancer cells, the expression and activation of kinesin and dynein, which regulate lysosome movement, change dynamically to regulate the locations of lysosomes within cells. The spatial distribution of lysosomes is involved in cancer cell metastasis and drug resistance [49, 50].

Therefore, lysosome functional changes and spatial distribution changes are both closely related to cancer development and progression [36, 51]. Comprehensive examination of specific lysosome changes in cancers and in-depth investigation of the intrinsic molecular mechanisms underlying these changes can yield a more comprehensive understanding of the dynamic changes in carcinogenesis and cancer development, thereby providing more opportunities for cancer diagnosis and targeted lysosomal treatment.

## Lysosome functional status and cancer development and progression

As mentioned above, lysosomes participate in a variety of life activities in normal cells. Likewise, lysosomes play crucial roles in cancer development and progression (Table 1).

**Table 1:** Lysosomes play crucial roles in cancer development and progression

Lysosome-associated biological processes or molecules	Mechanism	Effect on cancer
Endocytosis Macropinocytosis Phagocytosis	Recycling of exogenous materials provides energy, lipids and amino acids for cancer cells. H-rasG12V can stimulate membrane ruffling and pinocytosis. Macropinocytosis is a feature of RAS-transformed cells and can provide energy or metabolite precursors and scavenge lipids in Kras-driven cancer cells, such as pancreatic cancer and lung adenocarcinoma	Dysregulating cellular energetics
Autophagy (Atg5, Atg7, ULK1, BECN1)	Intracellular materials or entire organelles can be delivered to the lysosome for catabolism to provide energy or metabolite precursors to support a transformed phenotype. Failure to clear damaged mitochondria will impair tumor progression. In Kras-Lkb1 mutant lung tumors, inhibition of lysosomal activity causes mitochondrial defects and cancer cell death. Autophagy genes are important in cancers. The deletion of autophagy gene Atg5 or Atg7 inhibits the development of invasive cancers in a mouse model of pancreatic cancer driven by activation of oncogenic KrasG12D. In renal cell carcinoma and soft tissue sarcoma, MiT/TFEB escape surveillance by mTORC1 and become constitutively localized to the nucleus to drive gene expression programs for lysosome biogenesis and autophagy	Dysregulating cellular energetics
mTORC1	The lysosome plays an important role in nutrient sensing. When nutrients are deficient, cancer cells can inhibit the activation of mTORC1 on the lysosomal membrane and enhance autophagy to provide energy for themselves. It has been shown that the GATOR1 complex, a GAP for RagA/B, is deleted in human cancers. Lysosomal signaling can stimulate transcriptional programs that regulate lipid catabolism under starvation conditions	Dysregulating cellular energetics
Lysosome biogenesis Hydrolase Lysosomal peripheral localization	Premalignant cells evade oncogene-induced senescence to replicate indefinitely. Autophagy-dependent lysosomal processes can process senescence-associated chromatin fragments and maintain senescence-mediated tumor suppression. SV40 transformation, MYC expression, and mutant KRAS expression can increase the expression of cathepsins and glycosidases. Inactivation of p53 results in a lack of cathepsin activation. Lysosomal peripheral localization maintains cell membrane integrity and repair during cancer cell division	Promoting the immortalization of cancer cells
Cathepsins Cathepsin-activated MMPs Lysosome-derived exocytosis Lysosomal membrane proteins (LAMP-1) V-ATPase protein	Cathepsins (e.g., cathepsins B, S, and E) and cathepsin-activated MMPs can degrade extracellular matrix to promote local invasion. Lysosome-derived exocytosis of heparinase and cathepsins changes cell shape to promote invasion by cancer cells. Loss of cathepsin B in a mouse model of pancreatic cancer may decrease metastasis to the liver. Cathepsin L may also play a role in bone metastasis. LAMP-1 is highly expressed in highly metastatic tumor cells, especially metastatic colon cancer cells, indicating that lysosomal membrane proteins are important in cell adhesion and migration. The V-ATPase protein located in the lysosomal membrane can cause an acidic tumor microenvironment and promote the activity of hydrolases	Activating invasion and metastasis
Cathepsins Cathepsin-activated MMPs VEGFR2 recycling	Cathepsins and MMPs can promote angiogenesis by remodeling the extracellular matrix and basement membrane. The pro-form of cathepsin D stimulates mitogen-activated protein kinase signaling and angiogenic gene expression. It has been shown that cathepsin K has a role in neovascularization under hypoxic conditions by activating NOTCH1 signaling. The lysosome also functions in regulating endosome-to-plasma membrane recycling of VEGFR2	Promoting angiogenesis
Immune checkpoint recycling and degradation Exocytosis of secretory lysosome TRPMLs	Lysosomes play a crucial role in regulating tumor immunity. The expression, recycling, and degradation of immune checkpoints, such as CTLA-4 and PD-1, are dictated largely by lysosomal regulation. Secretory lysosomes can impact the function of NK cells and CTL by releasing granzymes, perforin, chemoattractants and so on. Lysosomal exocytosis can increase the surface area of the phagocytosing macrophage and promote engulfment of large particles. TRPMLs can impact immune function by regulating lysosomal exocytosis, endocytosis, and phagocytosis	Impairing antitumor immune response
Tumor antigen processing and presentation Autophagy	Lysosomes play an important role in tumor antigen processing and presentation. The deficiency of autophagy causes p62 (an autophagy adaptor) accumulation in HCC, which results in the generation of ROS through the dampening of NF-κB signaling. P62 accumulation, NF-κB signaling inhibition, and ROS generation can promote tumorigenesis by dampening dendritic cell function and impairing the antitumor immune response	Impairing antitumor immune response
Autophagy Lysosomal iron	Lysosomes can stimulate programmed cell death by the activation of autophagy or the lysosomal protease-dependent activation of caspases. The defect of lysosomes in cancer cells causes the inability to clear dead cell debris, which leads to the survival of neighboring cells. There is crosstalk between the autophagy and apoptosis pathways in cancer. The antiapoptotic protein BCL-2 can promote cancer cell survival by limiting autophagy and preventing autosis. The accumulation of iron in lysosomes creates favorable conditions for ROS formation by Fenton reactions to promote tumorigenesis	Resisting cell death
Lysosomal enzymes Glucocorticoid receptor Autophagy (ATG7, Beclin 1)	Lysosomes play an important role in tumor initiation stimulated by chronic inflammation. Cathepsin B can cleave trypsinogen-1, cause pancreatitis, and increase pancreatic cancer risk. Heparanase activated in the lysosome can degrade heparin sulfate proteoglycans and thereby regulate the activity of cytokines and growth factors such as TGF-β. Defects in autophagy genes ATG7 and Beclin 1 also cause chronic inflammation and promote spontaneous cancer of the lung, liver and lymphocytes	Tumor-promoting inflammation
Autophagy (Beclin, Atg5) Lysosomal integrity	Autophagy and lysosome activity participate in the maintenance of genome stability. Kidney cells isolated from Beclin 1 + / − Atg5 − / − mice show accumulation of p62 (an autophagy adaptor). Serial passage of these cells leads to alteration of cell ploidy and genomic instability. Because of deficient autophagic flux, cells cannot recycle nucleic acids, leading to nucleotide depletion and DNA damage. Cancer cells are usually aneuploid. Untransformed aneuploid cells show decreased autophagy, which correlates with the degree of karyotypic imbalance. A lack of lysosomal integrity causes leakage of DNAses from the lysosome and promotes tumorigenesis	Promoting genome instability
RTK recycling and degradation Autophagy Lysosome membrane	Lysosomes can regulate proliferative signaling through the endocytosis, degradation and recycling of RTKs. Autophagy pathways can also regulate proliferative signaling through the uptake and degradation of intracellular RTK signaling mediators. The activation of mTORC1 depends on its correct position to the lysosome membrane surface	Sustaining proliferative signaling
M6P/IGF2R	M6P/IGF2R is responsible for the proper trafficking of lysosomal hydrolase. Lysosomal hydrolase can be delivered to lysosomes, where signaling factors are processed that play a role in tumor suppression. The expression of M6P/IGF2R in hepatocellular carcinoma is decreased. M6P/IGF2R is also mutated in colon, breast, prostate, kidney, and lung cancers. Mutant M6P/IGF2R leads to mislocalization of hydrolase and fails to activate TGF-β, a class of conserved cytokines that suppress cell proliferation	Sustaining proliferative signaling

### Lysosomes and cancer energy metabolism

Continuous proliferation requires a sufficient energy supply and raw materials for macromolecular synthesis. The uptake and decomposition of extracellular glycoproteins and glycolipids and the recycling of intracellular substances are pathways for cancer cells to obtain carbohydrates, lipids and amino acids [52]. The extracellular substances obtained by phagocytosis, endocytosis and macropinocytosis can be further delivered to lysosomes to generate nutrients through lysosomal degradation. Moreover, through autophagy, intracellular substances are degraded into the nutrients and energy required by cancer cells. Although the microenvironment of cancer cells is poor, another core function of lysosomes in cancer cells is to provide energy and metabolize precursors through the recycling of endogenous or exogenous macromolecules [53, 54]. In KRAS-driven lung cancer and pancreatic ductal adenocarcinoma cells, lysosomes can degrade substances that are recycled from the extracellular and intracellular environments to provide materials for cancer cell growth [55], and prevent AMP accumulation, energy crisis, and fatal nucleotide degradation [56]. As mentioned above, lysosomes play a key role in cellular nutrient sensing. Studies have found that some amino acids can be directly sensed and bound by molecules such as amino acid receptors and transporters in the plasma membrane and cytoplasm as signal molecules; these amino acids can also be perceived by lysosomes [54]. mTORC1 is a highly conserved kinase complex in eukaryotic cells that can sense and integrate stimulation information such as energy and nutrient status to regulate cell growth and autophagy. When nutrients are lacking in cancer cells, MiT/TFE family of transcription factors can escape mTORC1-mediated negative regulation and locate in nucleus, thereby allowing cancer cells to maintain the activation of mTOR signaling and autophagy at the same time [57]. The activation of autophagy ensure efficient recycling of cellular material. This mechanism is associated with a variety of cancer metabolic activities. In cancers such as pancreatic ductal adenocarcinoma, renal cell carcinoma and non-small cell lung cancer, TFE3/TFEB and other transcription factors are activated to promote lysosomal biogenesis and functional activation, thereby maintaining steady-state metabolism in cancer cells and further promoting cancer malignancy [58–60]. This signal transduction mechanism not only upregulates lysosome biosynthesis but also increases autophagy to help cells cope with nutritional stress. The deletion of GATOR1 has been observed in human cancers and suggests that aberrant mTORC1 nutrient sensing plays a crucial role in cancers [25].

### Lysosomes maintain cancer cell proliferation

Malignant cells must avoid oncogene-induced senescence (OIS) to achieve continuous proliferation [61]. The role of OIS in the inhibition of carcinogenesis is very important and involves gene expression at cell cycle checkpoints and activation of the aging-related secretory phenotype [62–65]. Interestingly, a large number of lysosome-specific phenotypes can be observed in senescent cells, including upregulated lysosomal gene expression and increased lysosome number/volume [66, 67]. The metabolic activity of senescent cells was originally thought to be lower than that of proliferating cells; however, studies have shown that the metabolism of senescent cells is actually hyperactive and that the corresponding changes in lysosomes may provide a greater material basis for senescent cells [68–71]. During the OIS process, cellular oxidative metabolism increases, which is often associated with changes in chromatin structure, such as senescence-related heterochromatin. Heterochromatin foci can be extruded from the nucleus and enter the cytoplasm. Cancer cells degrade these cytoplasmic chromatin fragments by increasing the level of autophagy through increased lysosome synthesis, thereby maintaining the function of cancer cells and slowing aging [72]. Interestingly, the activation of proto-oncogenes or the absence of tumor suppressor genes can induce cell proliferation and induce changes in lysosome synthesis. In SV40-mediated immortalized transformed cells, molecular events such as MYC gene amplification and overexpression and KRAS mutant expression can increase the expression of lysosome catalase and glycosidase (including cathepsin D and cathepsin E) [73], suggesting that the expression of oncogenes can increase the number of lysosomes and enhance their functional state. In KrasG12D-driven lung tumor cell, the deletion of Atg5 or Atg7 reduces cell proliferation and tumor burden, suggesting that this is due to impaired autophagy. Atg7 deficiency can activate p53, which contributes to tumor suppression [74, 75]. Atg7 deficiency also reduces intiation, proliferation and development of melanoma, prostate cancer and colorectal cancer [76–78]. FIP200 is an essential autophagy protein to initiate autophagosome formation and the ablation of FIP200 can diminish the tumor-initiating properties of breast cancer stem cells [79]. Lysosomal calcium homeostasis can affect tumor proliferation. TRPML-2 knockdown can inhibit cell viability and proliferation, affect the cell cycle, promote apoptotic cell death in glioma cell lines. The mRNA and protein levels of TRPML-2 have been shown to increase with pathological grade [80]. The above results indicate that during the process of unregulated cancer cell proliferation, lysosomes exhibit increased biosynthesis and an enhanced functional status, which promotes intracellular substance circulation and the degradation of harmful intracellular byproducts, thereby maintaining cancer cell proliferation.

### Lysosomes promote cancer invasion and metastasis

Invasion and metastasis are the most prominent biological characteristics of malignant cancers and are also the leading causes of death among patients. Epithelial–mesenchymal transition (EMT) plays a critical role in cancer metastasis by enabling epithelial cells to acquire motility and invasiveness, which are characteristic of mesenchymal cells [81]. Autophagy plays an important role in cancer invasion and metastasis. Studies have found that autophagy is activated under adverse conditions, such as hypoxia and the accumulation of acidic metabolic products. Cells can use autophagy to degrade epithelial-derived molecules such as E-cadherin to induce EMT, thereby enhancing cancer cell invasiveness and metastasis [82, 83]. In vitro, EMT-inducing factors can downregulate the expression of E-cadherin on the plasma membrane of cancer cells by promoting the degradation of E-cadherin in lysosomes and inhibiting recycling, which suggests that the lysosomal degradation pathway promotes invasion and metastasis [84]. In addition, some metastasis suppressors, such as NM23-H1, can promote breast cancer invasion through lysosomal degradation [85]. Autophagy also supports cancer invasion and metastasis by promoting disassembly of cell–matrix FAs. This process was mediated by the interaction of processed LC3 with paxillin, a key FA component [86]. Autophagy-dependent secretion of the proinvasive cytokine, such as IL6, also promotes cancer invasion [87].

Degradation and modification of the ECM are necessary conditions for cancer invasion and metastasis [88, 89]. The release of lysosomal hydrolases, such as cathepsin, plays an important role in this process. TRPMLs and TPCs can affect the functional status of lysosomes and promote tumor invasion and metastasis by regulating lysosomal calcium homeostasis [14]. TRPML1-mediated lysosomal calcium release can promote TFEB nuclear translocation and increase lysosome biogenesis and autophagy. The activation of TPCs also promotes TFEB nuclear translocation. In the human hepatocellular carcinoma cell line HepG2, tetrabromobisphenol A (TBBPA) activates TRPML1, which promotes the release of lysosomal calcium and the nuclear translocation of TFEB and increases lysosomal exocytosis. Cancer cells then secrete cathepsins through lysosomal exocytosis [15]. Cathepsin can act directly or through the activation of matrix metalloproteinases (MMPs) to degrade and remodel the ECM, thus enhancing the invasion and metastasis of cancer cells. A study on a mouse model of pancreatic cancer found that the absence of cathepsin B reduced the probability of liver metastasis and prolonged the survival time of cancer-bearing mice [90]. Cathepsins B, S, and E are all involved in invasion and metastasis in various cancers [91–93]. Silencing TPC1 and TPC2 can reduce the adhesion and migration of invasive tumor cells. The inhibition of TPCs leads to the accumulation of integrins in endocytic vesicles and to impaired formation of leading edges. Alternatively, the inhibition of TRPMLs or TPCs may affect EGFR recycling and possibly delay or prevent cancer cell migration and/or proliferation [94, 95]. In addition, studies have confirmed that various lysosomal proteins, such as lysosome-associated protein-1 (LAMP1) [96, 97], LAMP3 [98, 99] and LAPTM4BP [100], are highly expressed in many malignant cancers, including melanoma, lung cancer, breast cancer and liver cancer, and that such high expression is associated with invasion and metastasis. LAMP-1 is abundant on the cell surface of highly metastatic cancer cells, especially metastatic colon cancer cells, which suggests that lysosomal proteins are important in cell adhesion and migration [101]. Researchers have examined the sensitivity of bladder cancer cell lines with different invasive potentials to the lysosomal inhibitors chloroquine (Cq) and bafilomycin and found that highly invasive bladder cancer cells were more sensitive to Cq and bafilomycin, while the invasive ability of Cq-resistant cells selected by screening highly invasive cells was significantly decreased [102]. These results suggest that lysosomes can be used as potential therapeutic targets in metastatic cancers.

### Lysosomes promote cancer angiogenesis

Angiogenesis has an important impact on cancer growth, invasion and metastasis. Remodeling of the ECM and vascular basement membrane is essential for initiating angiogenesis and vascular sprouting [103, 104]. The lytic granules cleaved by lysosomal exocytosis can destroy vascular basement membrane components at physiological pH [105]. Studies have shown that cathepsins D, B, S, K and L all play roles in promoting angiogenesis. On the one hand, activation of MMPs by cathepsin can mimic angiogenesis; on the other hand, cathepsin can directly act as a cytokine to stimulate the proliferation of vascular endothelial cells, thereby playing a role in promoting angiogenesis [106]. In addition, under anoxic conditions, cathepsin K can play important roles in angiogenesis through the activation of Notch homolog 1, translocation-associated (NOTCH1) signaling. Cathepsin K knockdown in endothelial cells results in reduced angiogenesis [107]. In addition, lysosomes also play a role in endothelial cell migration factor regulation. Rab GTPase is essential for angiogenesis and participates in the endosomal recycling of vascular endothelial growth factor receptor 2 (VEGFR2) [108]. Genetic deletion of Rab4a and Rab11a and the inhibition of lysosome activity by chloroquine can lead to defects in VCl2 lysosomal-plasma membrane recycling and inhibition of endothelial cell migration [108]. Lysosomal calcium homeostasis is associated with angiogenesis. The blockade of TPCs can inhibit VEGF-induced neoangiogenesis, which is mediated by TPC2-dependent calcium signaling. The inhibition of signaling pathways involving VEGFR2, NAADP, TPC2, and Ca2+ release from acidic stores can greatly reduce the activation of VEGFR2 downstream targets, which would then block angiogenesis, in both in vitro and in vivo models [109]. In-depth studies on the internal mechanisms and key molecules of lysosomal-regulated angiogenesis are currently lacking. Further studies on the specific roles of lysosomes in cancer angiogenesis may lead to the development of new anti-angiogenesis therapeutic strategies.

### Lysosomes and cancer immunity

In recent years, the great success of immune checkpoint therapy has confirmed the role of the immune system in cancer treatment [110]. It has been shown that lysosomes can serve as a major destruction location for immune checkpoint molecules, as secretory lysosomes can temporarily store immune checkpoint proteins, such as CTLA-4, PD-L1, TIM-3, CD70, CD200, and CD47 [111]. Studies have shown that CTLA-4 is a transmembrane T cell inhibitory protein mainly located in the plasma membrane and cytoplasm; however, attachment to the plasma membrane is important for CTLA-4 to perform its functions [112]. CTLA-4 expression is largely regulated by lysosomes. On the one hand, lysosomes degrade CTLA-4; on the other hand, lysosomes are responsible for CTLA-4 transport to the plasma membrane. CTLA-4 can bind to activator protein 1 (AP1) and AP2 [113, 114] to promote its transport to lysosomes for degradation. In addition, CTLA-4 can enter the cytoplasm for lysosomal degradation via endocytosis [115, 116]. Lysosomes containing CTLA-4 can be transferred to the T cell receptor (TCR), which subsequently secretes CTLA-4, increasing cell surface CTLA-4. After tyrosine phosphorylation, CTLA-4 remains on the cell surface [117, 118]. Therefore, the expression of other inhibitory receptors in T cells (e.g., PD-1) can be confidently assumed to also be similarly regulated by lysosomes; however, the role of lysosomes in this process is still unclear.

Secretory lysosomes, also known as lytic granules, contain proapoptotic granzymes and perforin and can also participate in the regulation of immune cell functions. Natural killer (NK) cells and cytotoxic T lymphocytes (CTLs) play a crucial role in immunity, as they are responsible for the elimination of both virally infected and tumorigenic cells. The clearance of target cells is dependent on the regulated exocytosis of secretory lysosomes, which can deliver proapoptotic granzymes and perforin to target cells [119]. Upon recognition of target cells, microtubules and actin filaments in CTLs are reorganized, which results in the polarization of the centrosome towards the immunological synapse (IS), which are formed with the target cells. Rab7 can interact with Rab7-interacting lysosomal protein (RILP) to recruit dynein to secretory lysosomes, which mediate minus-end-directed movement of secretory lysosomes to IS. Then, the contents in secretory lysosomes can be released, which leads to the destroy of target cells [120]. However, cancer cell autophagy may serve to intercept granzymes and perforin released by cytotoxic immune cells, blunting the efficacy of anti-tumor immune response [121, 122]. Impaired autophagy in breast cancer cells activates the immune response, IFN production and lymphocyte infiltration [79].

TRPMLs also play an important role in immunity [123]. When macrophages bind particles, the TRPML1 channel in lysosomes becomes activated and mediates Ca2+ release from lysosomes, which induces lysosomal exocytosis at the site of the phagocytic cup; this in turn increases the surface area of the phagocytosing macrophage and promotes the engulfment of large particles. TRPML1-mediated Ca2+ release is indispensable for phagosome maturation [124]. Macrophages can produce and secrete a variety of cytokines and chemokines after stimulation. Tumor-associated macrophages can be stimulated by IL-4, IL-10, or IL-13 and then migrate into tumor tissue, where they perform protumorigenic functions [125]. Recent findings have shown that the TRPML2 channel plays a crucial role in the release of chemokines as well as in the stimulation of macrophage migration [126, 127]. NK cell activity is regulated by the dynamic balance between activating and inhibitory signals, which determine whether NK cells kill the target cell. Major histocompatibility complex (MHC) class I molecules can be recognized by inhibitory receptors. The expression of MHC class I on virus-infected cells and tumor cells is decreased, which will be recognized by NK cells and promote the killing of these cells by NK cells. A process termed NK cell education describes the interaction between self-MHC and inhibitory receptors on NK cells, which calibrates NK cell effector capacities. TRPML1 participates in this process by regulating secretory lysosomes, granzyme B content, and the effector function of NK cells [128].

In summary, lysosomes in cancer cells are involved in various biological events affecting the development and progression of cancers. This finding provides useful clues for the diagnosis and treatment of cancers. Identification of the specific functions of lysosomes can help predict the prognosis of cancer patients and formulate individualized treatments. The functional status of lysosomes is closely related to their intracellular distribution. Understanding and exploring the lysosome distribution in cancer cells and the effects of different distributions on the development and progression of cancers can provide more comprehensive lysosome information and thus a theoretical basis for further individualized diagnosis and treatment strategies for cancer.

## The spatial distribution of lysosomes and cancer development and progression

Lysosomes exhibit different spatial distributions in cancer cells. In most cases, lysosomes are scattered in the cytoplasm, but some lysosomes are concentrated around the nucleus and can also be distributed in the plasma membrane. Studies have shown that in addition to the aforementioned functional status of lysosomes, the spatial distribution of lysosomes significantly affects the biological properties of cancer cells.

### Movement and spatial distribution of lysosomes

**Fig. 3:**
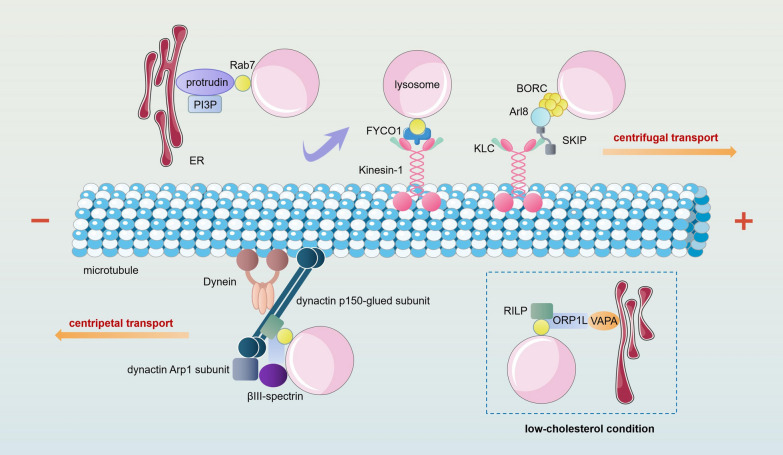
Movement and spatial distribution of lysosomes. Lysosomes can move towards the plus or minus ends of microtubules. The movement towards the plus end of microtubules is mediated by kinesins, of which the best characterized is kinesin-1, which is composed of two heavy chains (KHC) and two light chains (KLC). BORC can associate with lysosomes and recruit Arl8. SKIP binds to Arl8 through an N-terminal RUN domain and interacts with KLC through a WD motif in an unstructured region. Moreover, the ER-anchored protein protrudin binds simultaneously to Rab7 and PI3P to bridge the ER and lysosome. Protrudin then transfers lysosomes to the Rab7 effector FYCO1 and kinesin-1. The movement towards the minus end of microtubules is mediated by dynein. Lysosomal transport mediated by dynein is dependent on dynactin. The recruitment of dynein-dynactin to lysosomes is dependent on Rab7. RILP can link Rab7 to the dynactin p150-glued subunit. ORP1L forms a tripartite complex with Rab7 and RILP, promoting the association of βIII-spectrin with the dynactin Arp1 subunit. The interaction of ORP1L with Rab7–RILP and βIII-spectrin can activate dynein. The ability of ORP1L to engage is dependent on cholesterol levels. Under low-cholesterol conditions, ORP1L will bind VAPA, resulting in the dissociation of dynein-dynactin from lysosomes

Lysosomes are widely distributed in the cytoplasm. In nonpolarized cells, lysosomes are mainly concentrated in the microtubule-organizing center (MTOC) around the nucleus, but some peripheral lysosomes can reach or protrude through the plasma membrane [129]. In cells with obvious polarity, such as neuronal cells, lysosomes are distributed in various parts of the cytoplasm, including soma, axons and dendrites [130]. Lysosomes in the cytoplasm can move along the microtubules in and around the cell center. Movement towards the plus (centrifugal) and minus (centripetal) ends of microtubules is mediated by kinesin [131] and dynein [132], respectively (Fig. 3). Of course, this type of movement does not occur randomly and is often induced and regulated by specific conditions. For example, acidification of the cytoplasm leads to proliferation of the perinuclear lysosome population, and subsequent alkalization can promote the return of these lysosomes to a central location [133, 134]. Other stimuli, such as starvation and drug-induced apoptosis, can cause centripetal aggregation of lysosomes [134, 135].

In nonpolarized cells, the plus ends of microtubules are oriented towards the periphery of cells. In polarized cells such as neurons, the plus ends are oriented towards the ends of axons. Therefore, in these cells, lysosome transport from the cell center to the periphery (centrifugal transport) is mediated by the kinesins. However, in some polarized cells, such as the dendrites of neurons (microtubules with mixed orientations), microtubules can orient towards other directions [136, 137]. In these cases, both centrifugal and centripetal transports are mediated by the kinesisns. Lysosome movement towards the plus end of microtubules is mediated by the kinesin superfamily (KIF), including kinesin superfamily 1 (KIF5A, KIF5B and KIF5C) [138–140], kinesin superfamily 2 (KIF3) [141, 142], kinesin superfamily 3 (KIF1A and KIF1B) [143–145] and kinesin superfamily 13 (KIF2) [143, 146]. Kinesin-1 is the well-characterized kinesin involved in the transport of lysosom. Kinesin-1 is a heterotetramer composed of two heavy chains (KHC) and two light chains (KLC) that can be recruited to lysosomes by the multisubunit BLOC-1-related complex (BORC), the Arf-like small GTPase Arl8, the Arl8 effector SifA, and kinesin-interacting protein (SKIP) [138, 147, 148]. BORC is an octameric complex that can associate with the cytosolic face of lysosomes and recruit Arl8 [149]. Then, through an N-terminal RUN domain, SKIP binds to Arl8 and interacts with the tetratricopeptide repeat (TPR) domain of the KLC by a WD motif in an unstructured region. This process completes the linkage of lysosomes to kinesin-1 [138]. The coupling of late endosomes to kinesin-1 involves an alternative mechanism. The Rab7 and phosphatidylinositol 3-phosphate bind to ER-anchored protein protrudin to bridge the lysosome and ER. Protrudin then transfers lysosomes to the Rab7 effector FYCO1 and kinesin-1 [150]. Microtubule-associated proteins (MAPs) usually play inhibitory roles in lysosome movement. A representative example is that the central nervous system TAU proteins can inhibit kinesin superfamily 1-dependent lysosomal transport [151, 152]. However, other MAPs, such as ensconsin and DCLK1, may promote the recruitment and activation of kinesins to specific populations of microtubules [153, 154]. In addition, the activity of kinesin can be regulated by kinesin binding protein (KBP). Under the action of KBP, kinesin motor domain inactivation may occur [155].

Lysosomal movement can also occur towards the minus end of a microtubule, which is mediated by dynein. Currently, two types of dynein are believed to exist: axonemal dynein, which has a role in flagella and cilia, and cytoplasmic dynein, which functions in other types of cells. Cytoplasmic dynein is involved in lysosome movement and can move lysosomes from the plus end to the minus end of microtubules (centripetal). In nonpolarized cells, cytoplasmic dynein mediates lysosome transport from the periphery to the center (centripetal) [156]. In neurons, cytoplasmic dynein mediates centripetal lysosome transport, and due to the mixed directionality of dendritic microtubules, cytoplasmic dynein may simultaneously mediate centripetal and centrifugal lysosomal transport in dendrites [157]. Lysosomal transport mediated by cytoplasmic dynein is dependent on dynactin. The recruitment of dynein-dynactin to lysosomes is mainly mediated by Rab7. Rab7-interacting lysosomal protein (RILP) can link Rab7 to dynein-dynactin through the dynactin p150-glued subunit [158]. The oxysterol-binding protein-related protein 1L (ORP1L) forms a tripartite complex with Rab7 and RILP, which promotes the association of βIII-spectrin with the dynactin Arp1 subunit [158]. The interaction of ORP1L with Rab7-RILP and βIII-spectrin can activate dynein, and the ability of ORP1L to engage in this process is dependent on cholesterol levels [159]. Under low-cholesterol conditions, the OSBP-related domain of ORP1L will bind the vesicle-associated membrane protein-associated protein A (VAPA), which results in the dissociation of dynein-dynactin from lysosomes and causes dispersal of lysosomes to the cell periphery. In contrast, ORP1L does not interact with VAPA, and dynein-dynactin mediates the movement of lysosomes towards the cell center under high-cholesterol conditions. In addition, other Rab proteins, such as Rab9A, Rab34 and Rab36, the lysosomal Ca2+- sensors ALG-2, LAMP-1, and LAMP-2 and the transmembrane protein 106B (TMEM106B), are also involved in regulating the coupling of lysosomes to dynein-dynactin [36, 160–163].

Generally, intracellular substances do not move in a straight line, as transport routes are usually bidirectional and can change. These dynamics have been observed for mitochondria, peroxisomes, endosomes, lipid droplets, synaptic vesicle precursors, and viral particles [164]. Because kinesin and dynein are unidirectional motor proteins, the above dynamics suggest that kinesin and dynein play a synergistic rather than independent role in intracellular transport. In the axonal transport of vesicles containing the cellular mammalian prion protein (PrPC), kinesin-1 and cytoplasmic dynein can associate with PrPC vesicles to direct bidirectional movement. Kinesin-1 light chains (KLC1), kinesin-1 heavy chains (Kinesin-1C), and dynein heavy chain (DHC1) play a major role in this process. In addition to mediating anterograde transport, KLC1 and Kinesin-1C also activate retrograde movement of PrPC vesicles. The absence of KLC1 or Kinesin-1C results in bidirectional decreases and reduces velocity distributions in both directions. Similarly, decreasing DHC1 can also result in bidirectional movement inhibition. The above phenomenon shows that the activities of these motors are tightly coupled. Despite this, kinesin-1C and KLC1 are not required for the vesicle association of DHC1, indicating that a stable motor subunit population on vesicles is not affected by the presence or absence of other motors. Motor subunits are associated with stationary and moving PrPC vesicles. Thus, impairment of the reverse movement observed after removing a certain motor subunit is likely a result of coordination between Kinesin-1C and DHC1 activities, instead of resulting from structural changes to motor-cargo associations [164].

### The effect of the spatial distribution of lysosomes on cancers

Kinesins such as KIF11, KIF25 and KIF5b are overexpressed in most cancer cells [49, 165]. As mentioned above, these proteins can interact with microtubules to change lysosome localization. When these proteins are knocked down, cancer cells may exhibit cathepsin-dependent death and increased sensitivity to anticancer drugs such as siramesine [36]. These results suggest that kinesin-mediated changes in lysosomal distribution play an important role in ensuring cancer life activities and drug resistance.

Autophagy is a conserved and multipathway-mediated process for organelle degradation in eukaryotic cells [36]. Autophagy is a type of reactive cellular response to changes in the internal and external environment that plays an important role in cell survival and homeostasis [36]. First, an autophagosome is formed by wrapping proteins, organelles and other molecular particles under the membrane structure. Then, in the process of dynein-mediated centripetal lysosomal transport, lysosomes and autophagosomes fuse to form autophagic lysosomes, thereby completing the autophagy process [36]. Cardoso et al. found that autophagy relies heavily on lysosome distribution. Downregulation of KIF5b in human HeLa cervical cancer cells induces lysosome accumulation around the plasma membrane and autophagosome accumulation around the nucleus and suppresses the fusion of autophagosomes and lysosomes. These results suggest that lysosome movement and distribution regulate cancer cell autophagy [49].

The function of immune cells in cancer tissues also depends on the distribution of lysosomes. Immune cells such as mononuclear macrophages can recognize and phagocytose endogenous and exogenous substances and further degrade endocytosed substances in lysosomes. Studies have shown that changes in lysosomal distribution and movement affect the immune killing effect and degradation capacity of mononuclear macrophages [166]. In addition, lysosomes perform an important role in antigen presentation. In mature dendritic cells, antigenic peptides that bind to MHC-II molecules are released from lysosomes, and then are transported to the plasma membrane via lysosomal movements, where they are presented to CD4-positive T lymphocytes and activate T cells to perform the corresponding immune functions [167, 168]. In addition, the lysosomal distribution is closely related to the ability of CTLs and NK cells to clear viruses or cancer cells. CTLs form immune synapses and release the contents of lytic granules into their target cells. Not only specific cytotoxic mediators but also lysosomal cavities and membrane proteins are contained in the lytic granules; therefore, lytic granules are considered “lysosome-associated organelles” [169] or “secretory lysosomes” [170]. Interestingly, CTLs deliver cytotoxic granules based on the same mechanism as lysosomal localizaition. After binding to their target cells, lytic granules in activated CTLs move towards the microtubule-organizing center, which is located under the plasma membrane of the immune synapse [171]. This movement is mediated by the Rab7-kinesin complex [120]. The Rab27a-kinesin-1 complex mediates movement from the center of microtubule tissues to the terminals of immune synapses [172]. Interfering in this lytic particle movement can greatly reduce the killing ability of CTLs.

The movement of lysosomes towards the periphery of cells is necessary for cancer growth, invasion and metastasis. During cancer progression, lysosomes undergo dynamic changes in quantity, morphology, intracavitary pH, hydrolase content and intracellular distribution [106, 173]. The most notable change is lysosomal transport from the central to peripheral cytoplasm [174, 175]. This redistribution can be induced by changes in the cancer microenvironment, such as a reduction in pH [106, 176], or changes in the expression of genes that regulate lysosomal localization and movement during carcinogenic transformation processes (e.g., an increase in the KIF5B mRNA level in various cancer tissues). The expression of epidermal growth factor receptor 2 (HER2) in human breast cancer cells has been found to upregulate the expression of KIF5B and promote its binding to lysosomes [49]. In addition, studies have found that compared with stromal cells or benign prostatic hyperplasia tissue, Rab7 mRNA levels in prostate cancer cells are significantly downregulated [177]. Rab7 knockdown can cause lysosome redistribution and enhance the invasive ability of cancer cells. Second, lysosomal centrifugal transport is critical for the extracellular secretion of cancer cells and maintenance of plasma membrane integrity [178, 179]. The peripheral localization of lysosomes can maintain plasma membrane integrity and repair functions during rapid cell division [180], while hydrolases secreted extracellularly can degrade and remodel the ECM, rendering it more suitable for cancer cell movement and thus promoting cancer cell migration and invasion [181, 182]. During extracellular secretion, the transport of MMPs in lysosomes also contributes to the invasion and metastasis of cancer cells [183, 184]. In addition, after activation, integrins such as α5β1 can be anchored on the lysosomal membrane, which is mediated by Rab25, and be further transported to the plasma membrane through lysosomal movement, thereby regulating the adhesion and migration of cancer cells [185]. The above findings indicate that lysosomal transport is essential for cancer cell exocytosis, ECM degradation and cell adhesion and migration. Lysosomal localization and movement may be potential targets for cancer diagnosis and treatment [186].

## Conclusion and prospects

In summary, the functional status and distribution of lysosomes affect various malignant biological events in cancer cells and regulate the development and progression of cancers. Therefore, real-time monitoring and determining the functional status of lysosomes may facilitate the development of precise personalized treatment regimens. In addition, because lysosomes participate in mitogen signal transduction and immune escape processes, targeted lysosomal treatment may conceivably slow or inhibit the transformation of precancerous lesions into cancer. Furthermore, the functional status of lysosomes is related to the expression of important immune checkpoint receptors; this knowledge may help improve the effectiveness of cancer immune checkpoint therapy. Lysosomes can also degrade important connective proteins (e.g., E-cadherin) by autophagy and phagocytosis. Therefore, inhibiting the degradation of E-cadherin by lysosomes may be a new strategy for treating cancer metastasis.

Lysosomal distribution and movement are dependent on complex interactions between microtubule motor proteins and actin cytoskeleton structures. Although a series of molecules that regulate such interactions have been discovered, more such molecules may be discovered in the future. These molecules may have a very close relationship with cancer development and progression. The following outstanding issues should be addressed in future work: How do lysosomes in cancer cells switch between static and dynamic states and between centrifugal and centripetal movements? What are the roles of different lysosomal movements and different lysosomal distributions in cancers? How are lysosomal movements and distributions regulated in cancer cells through conditions such as nutrient availability, extracellular pH changes and cellular stress? What effects do lysosomal movement and distribution interventions have on lysosome function in cancer cells? Can cancers be treated by regulating lysosome distribution? Obtaining answers to these questions will not only help to clarify the mechanisms underlying the development and progression of cancers but also provide information for therapeutic interventions for cancers.

## Data Availability

Not applicable.

## Abbreviations

mTORC1: Mammalian target of rapamycin complex 1
CTLA-4: Cytotoxic T lymphocyte antigen 4
TCR: T cell receptor
TFEB: Transcription factor EB
rER: Rough endoplasmic reticulum
sER: Smooth endoplasmic reticulum
ECM: Extracellular matrix
GATOR: GAP activity towards Rags
OIS: Oncogene-induced senescence
EMT: Epithelial–mesenchymal transition
MMPs: Matrix metalloproteinases
LAMP1: Lysosome-associated membrane protein-1
Cq: Chloroquine
VEGFR2: Vascular endothelial growth factor receptor 2
KIF: Kinesin superfamily
SKIP: SifA and kinesin-interacting protein
MAPs: Microtubule-associated proteins
BORC: Multisubunit BLOC-1 complex
MTOC: Microtubule-organizing center
KBP: Kinesin binding protein
CTLs: Cytotoxic T lymphocytes
HER2: Epidermal growth factor receptor 2
M-6-P: Mannose-6-phosphate
TGN: Trans-Golgi network
ILV: Intraluminal vesicles
MVB: Multivesicular bodies
TRPML: Transient receptor potential mucolipin
TPC: Two-pore channel
CLEAR: Coordinated lysosomal expression and regulation
RTK: Receptor tyrosine kinases
SNARE: Soluble *N*-ethylmaleimide-sensitive fusion attachment protein receptor
EGFR: Epidermal growth factor receptor
PDGFR: Platelet-derived growth factor receptor
